# *Xiaoshuan* enteric-coated capsule alleviates cognitive impairment by enhancing hippocampal glucose metabolism, hemodynamics and neuroplasticity of rat with chronic cerebral hypoperfusion

**DOI:** 10.1038/s41598-018-25929-0

**Published:** 2018-05-10

**Authors:** Man-zhong Li, Yi Zhang, Hai-yan Zou, Ya-li Wang, Brian-Chi Yan Cheng, Lei Wang, Qiu-xia Zhang, Jian-feng Lei, Hui Zhao

**Affiliations:** 1 School of Traditional Chinese Medicine, Capital Medical University, Beijing, 100069 China; 2 Beijing Key Lab of TCM Collateral Disease Theory Research, Beijing, 100069 China; 3 Department of Pharmacology, Beijing University of Chinese Medicine, Beijing, 100102 China; 4 College of Professional and Continuing Education, The Hong Kong Polytechnic University, Hung Hom, Hong Kong, China; 5 Medical Imaging laboratory of Core Facility Center, Capital Medical University, Beijing, 100069 China

## Abstract

Chronic cerebral hypoperfusion (CCH) is identified as a critical risk factor of dementia in patients with cerebrovascular disease. *Xiaoshuan* enteric-coated capsule (XSECC) is a compound Chinese medicine approved by Chinese State Food and Drug Administration for promoting brain remodeling and plasticity after stroke. The present study aimed to explore the potential of XSECC to improve cognitive function after CCH and further investigate the underlying mechanisms. CCH was induced by bilateral common carotid artery occlusion (BCCAO) in rats. XSECC (420 or 140 mg/kg) treatment remarkably reversed BCCAO-induced cognitive deficits. Notably, after XSECC treatment, magnetic resonance angiography combined with arterial spin labeling noninvasively demonstrated significantly improved hippocampal hemodynamics, and ^18^F-FDG PET/CT showed enhanced hippocampal glucose metabolism. In addition, XSECC treatment markedly alleviated neuropathologies and improved neuroplasticity in the hippocampus. More importantly, XSECC treatment facilitated axonal remodeling by regulating the phosphorylation of axonal growth related proteins including protein kinase B (AKT), glycogen synthase kinase-3β (GSK-3β) and collapsin response mediator protein-2 (CRMP2) in the hippocampus. Taken together, the present study demonstrated the beneficial role of XSECC in alleviating BCCAO-induced cognitive deficits by enhancing hippocampal glucose metabolism, hemodynamics and neuroplasticity, suggesting that XSECC could be a useful strategy in cerebral hypoperfusion state and dementia.

## Introduction

Vascular cognitive impairment (VCI), which encompasses a large range of cognitive deficits including vascular dementia, has emerged as the second common cause of age-related cognitive impairment^1^. Chronic cerebral hypoperfusion (CCH), a common consequence of various cerebral vascular disorders, has been identified as a key etiological factor in VCI^2^. CCH can induce neuroplasticity damage, cerebral circulation abnormality, and hypoperfusion-related metabolic changes in various regions of the brain, including hippocampus, leading to an irreversible dysfunction in cognition^3,4^. Currently, many neuroprotective agents are commonly used to control the neuronal degenerative processes in patients with CCH^5^. However, these strategies are focused on preserving the neuronal functions, but ignore the hemodynamic and glucose metabolic disorders induced by CCH in clinic^6^. Given that the pathophysiology of CCH-induced cognitive impairment is complex, new therapeutic strategies with multi-targets are urgently needed to ameliorate cognitive impairment caused by CCH.

Complementary and alternative medicine is widely used to treat cerebrovascular disease. *Buyang Huanwu Tang* (, literally: *Yang*-tonifying and balance-restoring decoction BYHWT), a traditional Chinese medicinal formula, has been used for treating post-stroke impairments for hundreds of years in China^7^. It has been reported that BYHWT has remarkable cognition-enhancing effect with multiple mechanisms of action in vascular dementia rats induced by ischemic injury^8^. However, the clinical use of BYHWT is hampered by its inconsistent preparation, unstable quality, and variable standards. *Xiaoshuan* enteric-coated capsule (XSECC), a novel preparation of the BYHWT, was therefore developed in order to solve this problem. XSECC was authenticated and standardized on the basis of marker compounds in the Chinese Pharmacopoeia (Committee, 2015), and registered in the State Food and Drug Administration in China for the treatment of ischemic stroke. Previous study in our laboratory has shown that XSECC possesses therapeutic benefit on neurovascular coupling and dynamics in a rat model of permanent middle cerebral artery occlusion^9^. As neurovascular dysfunction plays an important role in the development of VCI, we speculate that XSECC may have potential cognition-enhancing effects after CCH. Therefore, in this study, we established bilateral common carotid artery occlusion (BCCAO) as a preconditioning stimulus to induce CCH rat model. As hippocampus played a central role in cognitive functions, we also investigated the possible efficacy of XSECC on cognitive functions, cerebral hemodynamics, and glucose metabolism, as well as histopathological changes in the hippocampus after BCCAO and explored the underlying mechanisms.

## Results

### XSECC improved the spatial learning and memory in BCCAO rats

**Figure 1:**
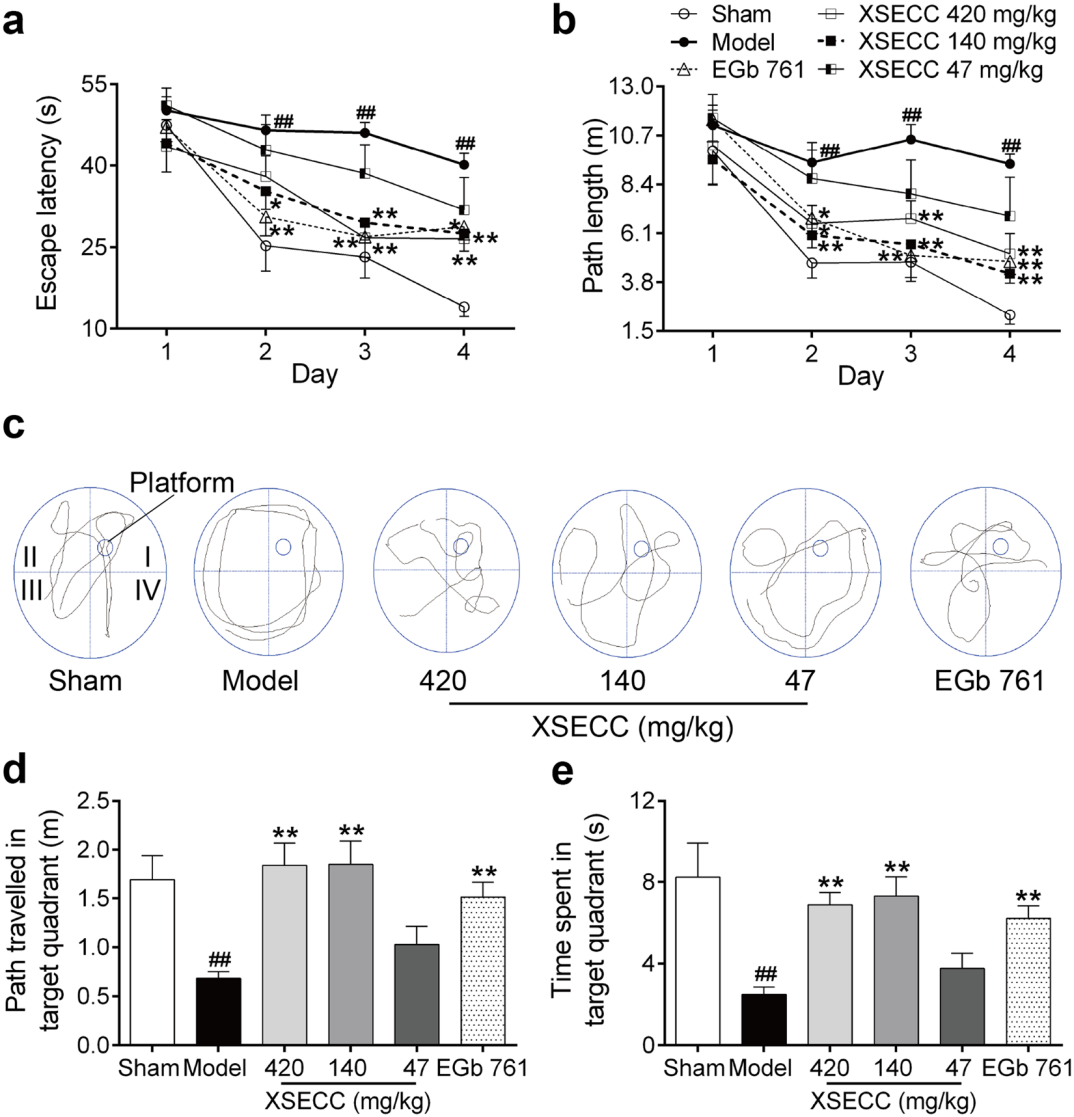
Effects of XSECC on Morris water maze performance deficits in BCCAO rats. (**a**) Escape latency and (**b**) path length of rats to find the emerged platform during the training trial (two-way ANOVA with repeated measures followed by Turkey’s *post hoc* tests). (**c**) Swimming traces of rats in the probe trial. The small cycle in quadrant I represents the platform and the big cycle represents the pool edge. (**d**) Path travelled and (**e**) time spent of rats in the target quadrant (quadrant I) during the probe test. ^##^*P* < 0.01 *vs*. sham group; *P < 0.05, **P < 0.01 *vs*. model group (one-way ANOVA followed by LSD’s *post hoc* test, XSECC 47 mg/kg group, n = 9–10).

Morris water maze test was performed to evaluate the hippocampus-related spatial learning and memory capacity of all rats. Repeated measures of ANOVA revealed a significant effect of day and treatment on the escape latency (day, *F* = 57.159, *P* < 0.001; treatment, *F* = 5.674, *P* < 0.001) and path length (day, *F* = 47.408, *P* < 0.001; treatment, *F* = 5.015, *P* = 0.001) in the hidden platform trial. Notably, a treatment × day interaction (*F* = 2.42, *P* = 0.003) was found on the escape latency. The results demonstrated improved performance over testing days in all group rats and treatment showed better neurobehavior than that of model group. *Post hoc* analysis showed that BCCAO rats without treatment took more time and covered longer swim paths to find the hidden platform on the 2^nd^, 3^rd^, and 4^th^ day as compared with the sham operated controls, indicating the learning capacity was impaired after BCCAO (*P* < 0.01, Fig. 1a,b). However, rats in the XSECC 140 mg/kg group found the platform faster on the 2^nd^ day than that in the model group (*P* < 0.01). XSECC (420 and 140 mg/kg) treatment rats showed reduced escape latency and path length in locating the hidden platform as compared with the model group (*P* < 0.01).

In the probe trial, the platform was removed. Figure 1c presented the swimming traces of rats in different groups. The model group rats covered shorter swim paths and displayed fewer time in the target quadrant (quadrant I), when comparing with the sham group rats (*P* < 0.01), indicating impaired memory retention after BCCAO. Notably, the rats treated with XSECC (420 and 140 mg/kg) displayed good spatial memory, as evidenced by a remarkably increased path and time spending in the previous platform location compared with the model group (*P* < 0.05 or *P* < 0.01). However, XSECC 47 mg/kg treatment exhibited no beneficial effects on these parameters compared with model group (Fig. 1d,e).

After Morris water maze test, BCCAO plus XSECC (420 and 140 mg/kg) group, sham surgery group, BCCAO model group were further divided randomly for MRI and histologic studies (six rats each), and western blot measurement (four rats each) to investigate the mechanisms underlying its anti-CCH effects.

### XSECC promoted hemodynamics in BCCAO rats

**Figure 2:**
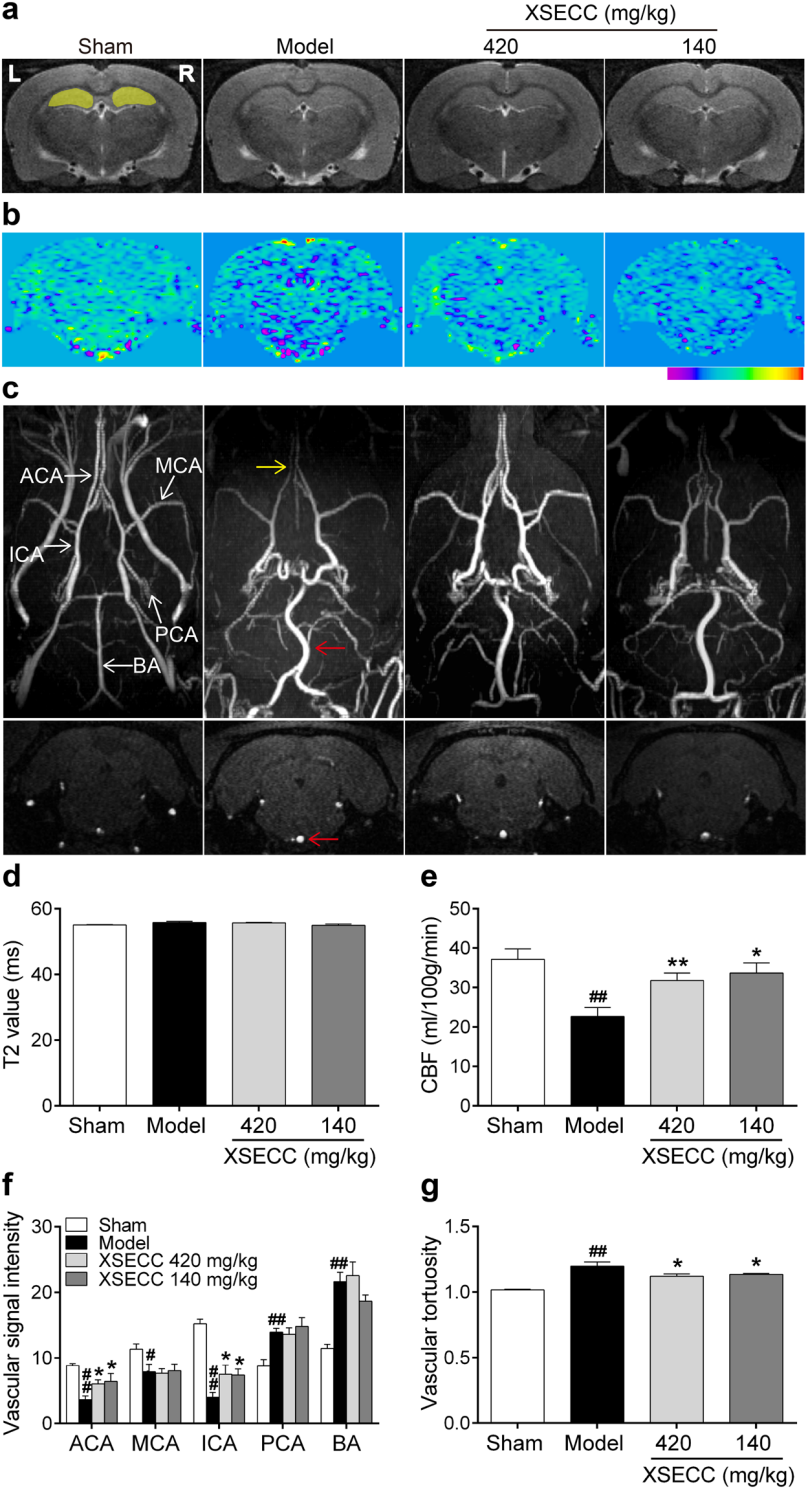
Effects of XSECC on cerebral vessels and cerebral blood flow in BCCAO rats. (**a**) Representative T2 relaxometry images of various group rats. The yellow cover on the sham group indicated ROIs of the bilateral hippocampus (R, right; L, left). (**b**) Typical CBF maps obtained from ASL at the hippocampus level (−3.8 mm relative to bregma). (**c**) Representative axial MIP maps (upper panel) and coronal data set images (lower panel) of MRA exhibited relatively distinguished cerebral vessels. ACA, MCA, ICA, PCA and BA were identified in the sham group map (ACA, anterior cerebral artery; MCA, middle cerebral artery; ICA, internal carotid artery; PCA, posterior cerebral artery; BA, basilar artery). The red arrow presented the signal-enhanced and tortuous BA, and the yellow arrow presented the weaken signal of ACA. Quantitation of hippocampal T2 values (**d**), hippocampal CBF (**e**), and vascular intensity of ACA, MCA, ICA, PCA, BA (**f**) and vascular tortuosity of BA (**g**). ^#^*P* < 0.05, ^##^*P* < 0.01 *vs*. sham group; *P < 0.05, **P < 0.01 *vs*. model group (one-way ANOVA followed by LSD’s *post hoc* test, n = 6 per group).

T2 relaxometry mapping was performed to examine the hippocampal structural abnormalities after BCCAO. As shown in Fig. 2a, T2 relaxometry images presented no abnormal signal after BCCAO, and there was no statistical difference of the hippocampal T2 values among all the groups (Fig. 2d).

Next, three-dimensional time-of-flight magnetic resonance angiography (3D TOF-MRA) was conducted to detect the morphological changes of intracranial vessels (Fig. 2c). The MIP maps of 3D TOF-MRA showed that the anatomical origin and course of bilateral anterior cerebral artery (ACA), middle cerebral artery (MCA), internal carotid artery (ICA), posterior cerebral artery (PCA) and BA were well displayed in the rats of sham group. Notably, BCCAO model group rats exhibited a weakened ACA signal (yellow arrow), tortuous and strengthened BA (red arrow). Quantitative data (Fig. 2f) showed that the MRA signal in the carotid artery system (ACA, MCA, ICA) decreased while the PCA and BA increased after BCCAO when comparing with the sham group (*P* < 0.05 or *P* < 0.01). XSECC (420 and 140 mg/kg) treatment markedly enhanced the signal intensities of ACA and ICA, and alleviated the tortuosity of BA compared with model group (*P* < 0.05). However, there was no statistical difference in the signal intensities of PCA and BA between the model and the XSECC (420 and 140 mg/kg) groups.

Additionally, arterial spin labeling (ASL) magnetic resonance imaging analysis showed that hippocampal CBF reduced in the model group compared with sham group (*P* < 0.01). However, XSECC (420 and 140 mg/kg) treatment markedly elevated the hippocampal CBF compared with model group (*P* < 0.05 or *P* < 0.01, Fig. 2e).

### XSECC enhanced glucose metabolism in the hippocampus of BCCAO rats

**Figure 3:**
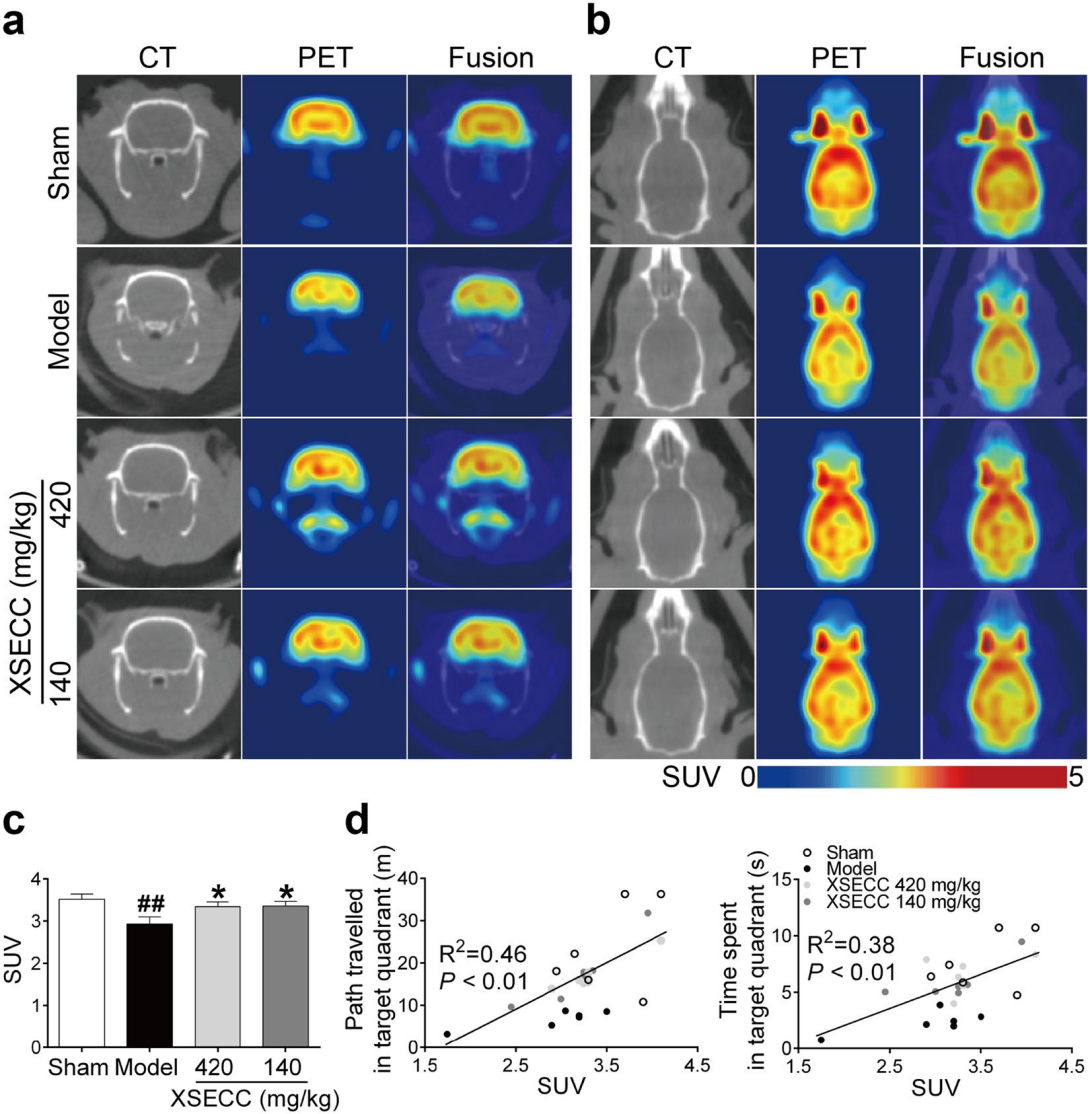
Effects of XSECC on glucose metabolism in BCCAO rats. ^18^F-FDG PET images of the (**a**) coronal and (**b**) axial planes. PET images are co-registered with the CT of the same animal to localize anatomically the PET signal. (**c**) Quantitative analysis of SUV of ^18^FDG in the hippocampus. ^##^*P* < 0.01 *vs*. sham group; *P < 0.05, **P < 0.01 *vs*. model group (one-way ANOVA followed by LSD’s *post hoc* test, n = 6 per group). (**d**) Person correlational analysis of the hippocampal SUV and Morris water maze parameters of the probe test. A positive correlation was found between the hippocampal SUV and path (Pearson R^2^ = 0.46, *P* = 0.0002), time spent in the target quadrant (Pearson R^2^ = 0.38, *P* = 0.0013) of the probe test (Pearson correlational analysis).

Glucose metabolism dysfunction in the hippocampus is closely related to the decline of cognitive function. Therefore, ^18^F-FDG micro PET/CT was used to detect the glucose metabolic changes in the hippocampus after BCCAO. Figure 3a,b exhibited typical coronal and axial PET/CT images of different group rats, respectively. Quantitative analysis indicated that the hippocampal standard uptake value (SUV) of ^18^F-FDG was significantly reduced in BCCAO model rats compared with sham rats (*P* < 0.01). However, XSECC (420 and 140 mg/kg) treatment markedly elevated the ^18^F-FDG accumulation in the hippocampus compared with model group (*P* < 0.05, Fig. 3c). Correlational analysis (Fig. 3d) revealed the SUV of ^18^F-FDG in the hippocampus was positively correlated with the path or time spent in the target quadrant of the probe test. These results suggested that the cognitive improvement of XSECC may be closely related to the glucose metabolic changes in the hippocampus.

### XSECC attenuated neural injury in the hippocampal CA1 region of BCCAO rats

**Figure 4:**
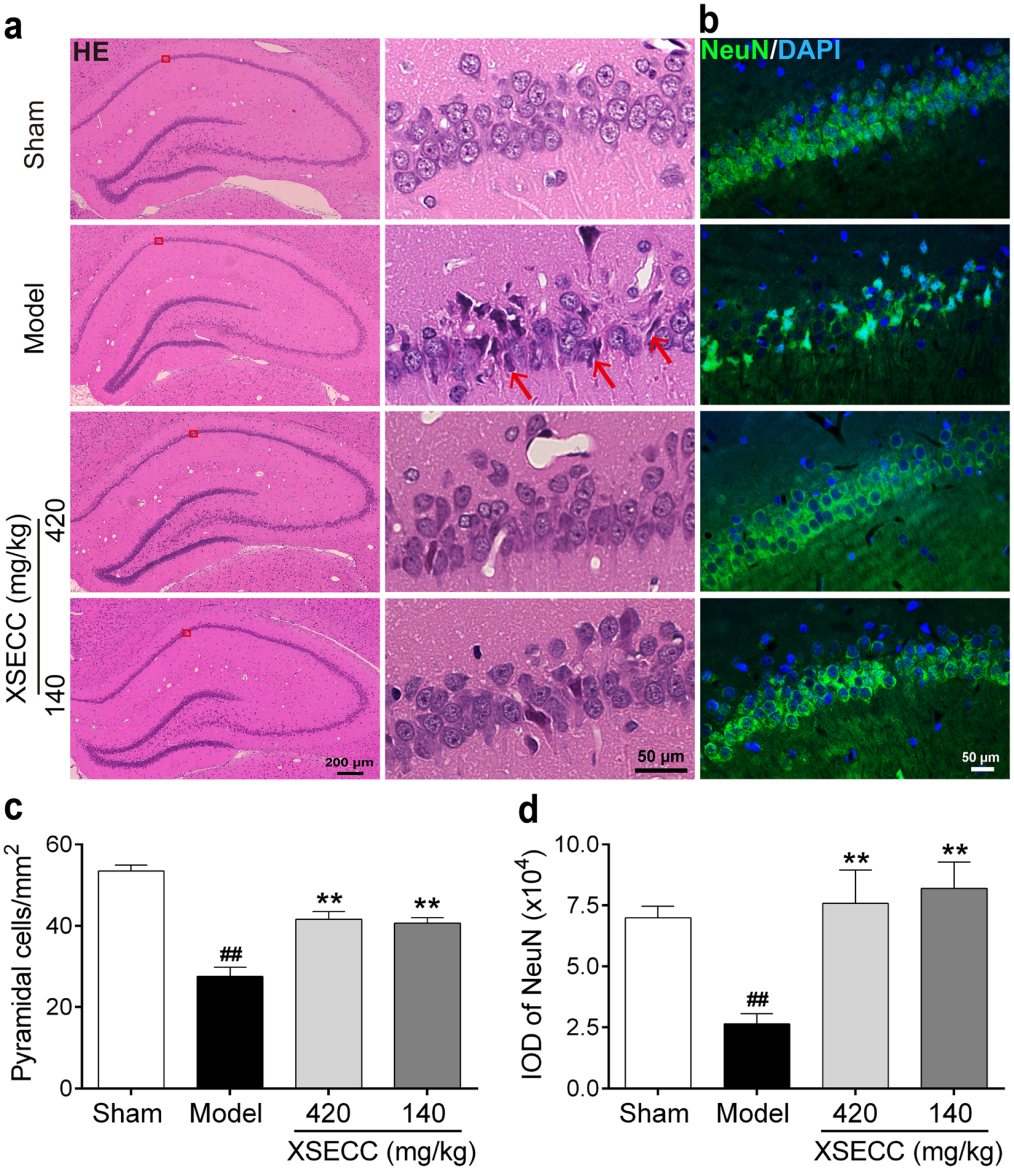
Effects of XSECC on neural injury in BCCAO rats. Four non-overlapping images (400× magnification) within hippocampal CA1 of each animal were taken for the analysis of HE staining and NeuN immunofluorescence respectively. (**a**) Representative HE staining of the unilateral hippocampus (Scale bar: 200 μm) and hippocampal CA1 area (Scale bar: 50 μm) from sham, model and XSECC group rats. Red rectangle represented the region of interest located in the CA1 area of hippocampus. The neurons are shrinkaged and dark-stained in the model group rats (red arrows). (**b**) Immunofluorescence staining of NeuN in the hippocampus CA1 area (Scale bar: 50 μm). (**c**,**d**) Analysis of pyramidal cells and NeuN expression in the hippocampal CA1 area respectively. ^##^*P* < 0.01 *vs*. sham group; **P < 0.01 *vs*. model group (one-way ANOVA followed by LSD’s *post hoc* test, n = 6 per group).

Hematoxylin and eosin (HE) staining was performed to examine the neuronal pathologically changes in the hippocampal CA1 regions. As shown in Fig. 4a, varying degrees of neuronal degeneration were found in hippocampal CA1 region after BCCAO as characterized by small pyramidal neurons appeared shrunken and stained darker with hematoxylin. However, XSECC (420 and 140 mg/kg) treatment attenuated the BCCAO-induced neural injury in the hippocampus. Quantitative analysis (Fig. 4c) revealed that the number of surviving cells in the hippocampal CA1 regions was significantly increased by treatment with XSECC (420 and 140 mg/kg) (*P* < 0.01).

To further confirm the HE founding, immunofluorescence staining of NeuN (a marker of mature neuron) was performed to identify neuronal injury. Figure 4b showed that the expression of NeuN-positive neurons in the hippocampal CA1 regions was markedly reduced in the model rats, as compared with the sham rats (*P* < 0.01, Fig. 4d). Treatment with XSECC (420 and 140 mg/kg) significantly increased the expression of NeuN-positive neurons in the hippocampal CA1 area, suggesting that XSECC protected neuronal cells.

### XSECC ameliorated axonal damage in the hippocampus of BCCAO rats

**Figure 5:**
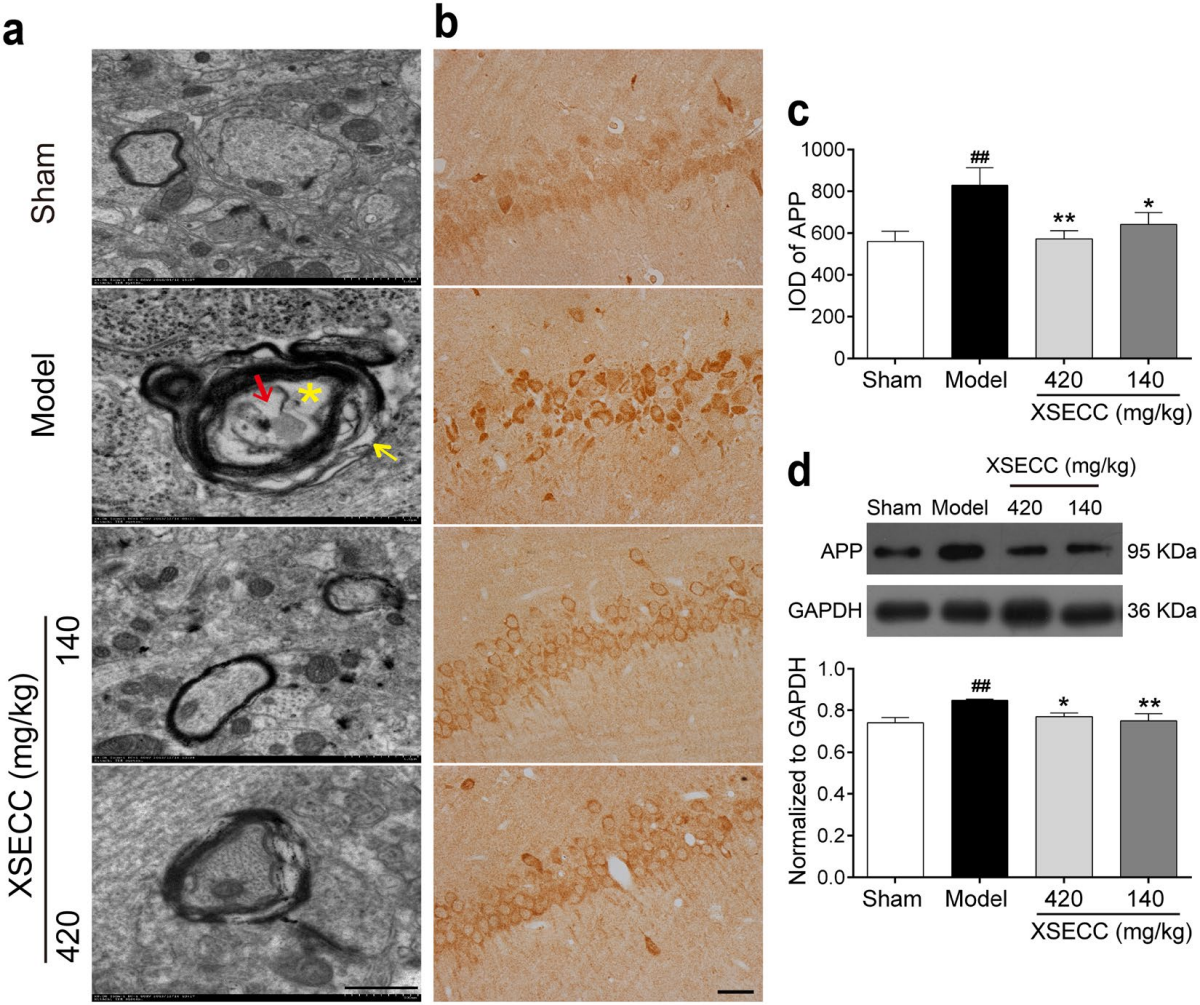
Effects of XSECC on axonal damage in BCCAO rats. (**a**) Typical TEM images of axonal ultrastructure from sham, model and XSECC group rats. (Yellow arrow represented loss of myelin sheath, yellow asterisk represented swollen axon, and red arrow represented degenerated axon, scale bar = 100 nm, n = 2 per group). (**b**) Immunohistochemistry staining of APP in the hippocampus CA1 area (scale bar = 50 μm, four sections per animal). Immunohistochemical (**c**) and western blot (**d**) analysis of APP protein expression in the hippocampus (n = 6 per group). ^##^*P* < 0.01 *vs*. sham group; *P < 0.05, **P < 0.01 *vs*. model group (one-way ANOVA followed by LSD’s *post hoc* test).

Transmission electron microscopy (TEM) was used to observe the ultrastructural alterations of axon. BCCAO rats showed axon swelling and myelin sheath decompaction in the hippocampus CA1 region (Fig. 5a). While XSECC (420 and 140 mg/kg) treatment partially improved axon swelling and myelin degeneration. Next, the protein expression of amyloid precursor protein (APP) was detected to evaluate the axonal injury in the hippocampus. The immunohistochemical staining and western blot results showed that the protein level of APP was significantly increased in the hippocampus of the BCCAO model group when compared with sham group (*P* < 0.01). However, XSECC (420 and 140 mg/kg) treatment markedly reduced the expression level of APP compared with model group (*P* < 0.05 or *P* < 0.01, Fig. 5c,d).

### XSECC improved neuronal plasticity in the hippocampus of BCCAO rats

**Figure 6:**
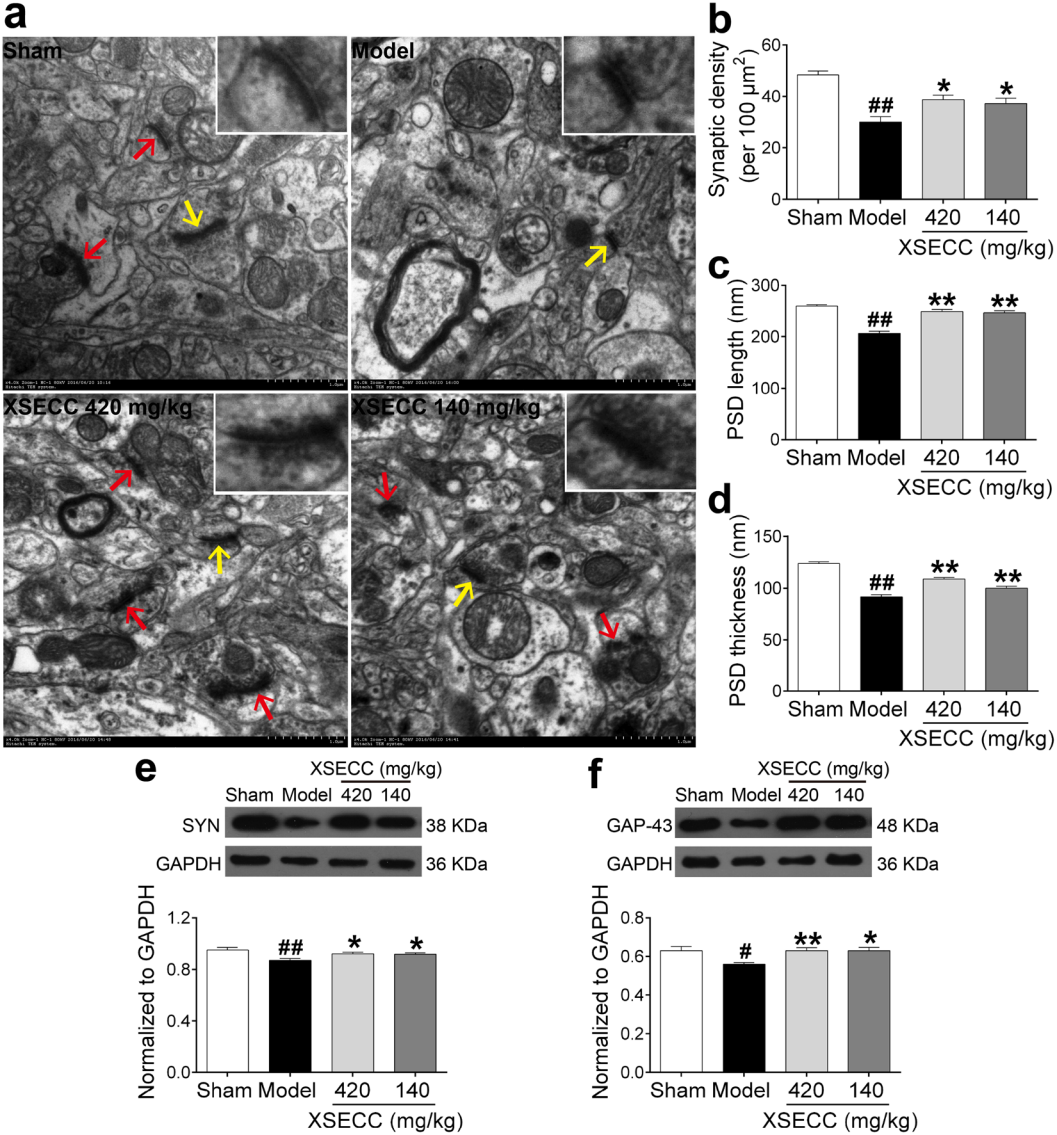
Effects of XSECC on neuronal plasticity in BCCAO rats. Twenty random images (8000×) were taken for the analysis of synaptic density and another twenty high magnification images (20000×) were taken for the analysis of PSD. (**a**) Typical TEM images of synaptic ultrastructure from sham, model and XSECC group rats (Scale bar = 500 nm, n = 2 per group). Synapses were identified by arrows and a magnified synapse (from the synapse indicated by yellow arrow) was displayed on the top right corner of the TEM images. Quantitation showed synaptic density (**b**), PSD length (**c**) and thickness (**d**) in the model group rats were significantly elevated by XSECC. Western blot analysis of the SYN protein expression (**e**) and GAP-43 protein level (**f**) in the hippocampus (n = 6 per group). ^#^*P* < 0.05, ^##^*P* < 0.01 *vs*. sham group; *P < 0.05, **P < 0.01 *vs*. model group (one-way ANOVA followed by LSD’s *post hoc* test).

To investigate whether XSECC has potential to promote hippocampal neuroplasticity after BACCO, the expressions of SYN and GAP-43, which were reliable markers of neuroplasticity^5,10^, were examined. As shown in Fig. 6e,f, the expression levels of SYN and GAP-43 in the hippocampus were significantly down-regulated after BCCAO compared with sham group (*P* < 0.05 or *P* < 0.01). However, XSECC (420 and 140 mg/kg) treatment markedly up-regulated the expression levels of SYN and GAP-43, when comparing with model group (*P* < 0.05 or *P* < 0.01).

Furthermore, the ultrastructural changes of synapses in the rat hippocampus CA1 region were observed by TEM after BCCAO. In the sham group rats, the presynaptic and postsynaptic membranes were clear and exhibited integrate lines with abundant postsynaptic density (PSD) (Fig. 6a). However, the synaptic ultrastructure was indistinct and synaptic density was evidently reduced in the model group rats (*P* < 0.01). In addition, some of the structural parameters of synaptic interface were altered induced by BCCAO, including a decreased length and thickness of PSD in the hippocampal CA1 area of model group rats (*P* < 0.01). XSECC (420 and 140 mg/kg) treatment not only elevated synaptic density in the hippocampus, but also increased PSD length and thickness compared to model group (*P* < 0.05 or *P* < 0.01, Fig. 6b–d).

### XSECC regulated the protein expressions of AKT/GSK-3β/CRMP2 pathway in the hippocampus of BCCAO rats

**Figure 7:**
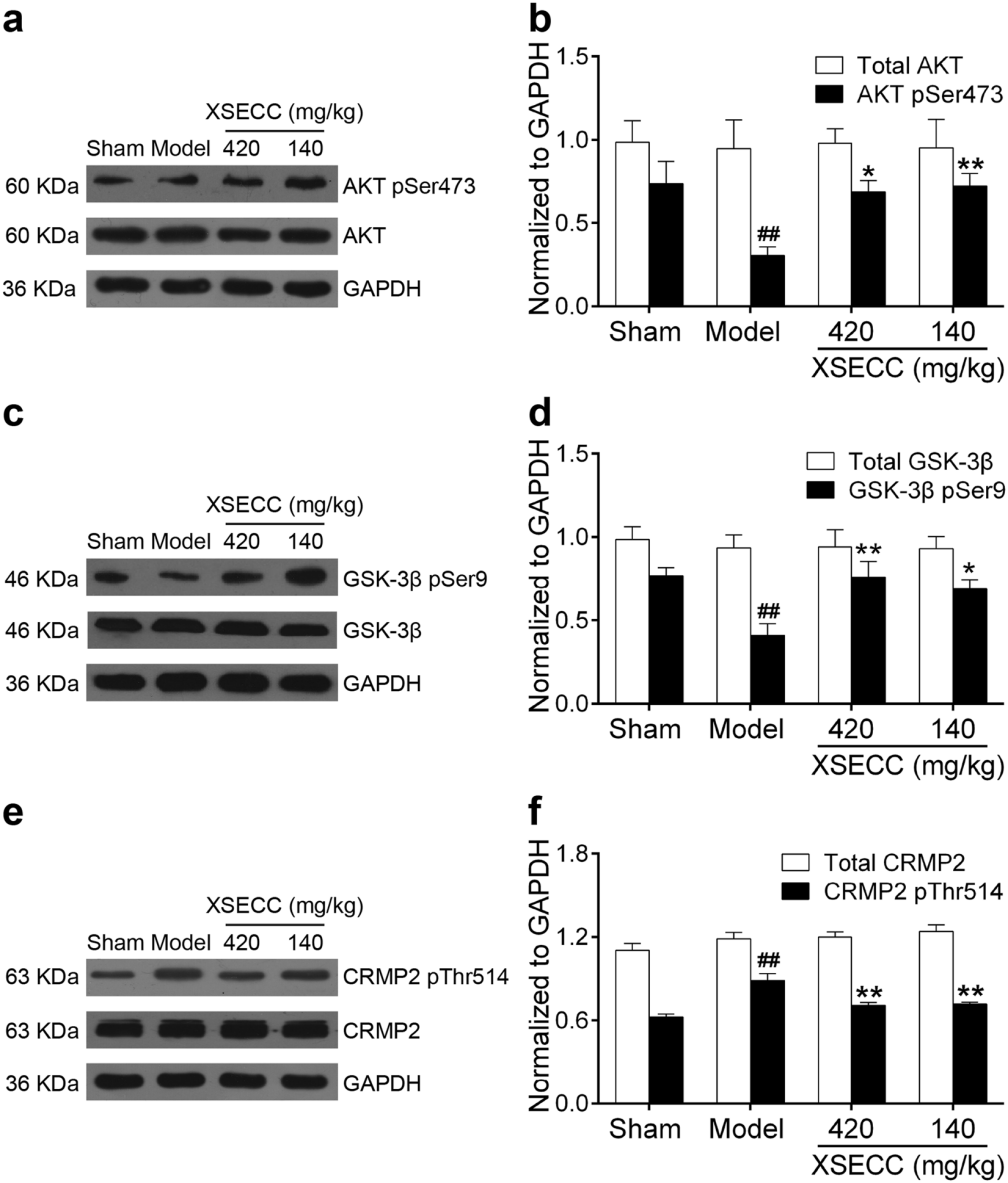
Effects of XSECC on the protein expressions of AKT/GSK-3β/CRMP2 signaling pathway after BCCAO. Protein and phosphorylation protein level of AKT (**a**,**b**), GSK-3β (**c**,**d**) and CRMP2 (**e**,**f**). ^##^*P* < 0.01 *vs*. sham group; *P < 0.05, **P < 0.01 *vs*. model group (one-way ANOVA followed by LSD’s *post hoc* test, n = 6 per group).

Since AKT/GSK-3β/CRMP2 signaling pathway plays an important role in axonal restoration after BCCAO, we further explored the effect of XSECC on the AKT/GSK-3β/CRMP2 pathway in the hippocampus of BCCAO rats. As shown in Fig. 7f, the phosphorylation protein level of CRMP2 was markedly increased after BCCAO (*P* < 0.01), while XSECC (420 and 140 mg/kg) treatment significantly decreased the phosphorylation level of CRMP2 (*P* < 0.01). Next, some effectors of the upstream CRMP2 were also examined. Figure 7b,d showed that the expression of phosphorylated AKT and GSK-3β was significantly decreased after BCCAO (*P* < 0.01). Treating rats with XSECC (420 and 140 mg/kg) markedly increased the phosphorylation level of AKT and GSK-3β (*P* < 0.05 or *P* < 0.01). In addition, there was no difference in the total AKT, GSK-3β or CRMP2 protein expression among all groups.

## Discussions

CCH is recognized as a pathological source for induction of dementia, which is illustrated by the cognitive decline and memory impairment^11–13^. In view of the pathological role of CCH in dementia, BCCAO rat model has been established as a procedure to investigate the effects of XSECC on cognitive dysfunction and neurodegenerative processes^14^. Since the hippocampus plays a central role in processing and consolidating the spatial-temporal context for memories^6^, we evaluated the cognitive-enhancing effect of XSECC using the Morris water maze, which is the most frequently used tests to measure the hippocampus-related spatial learning and memory in rats. In line with previous reports^15,16^, defective learning ability of rats represented by impaired ability to find the hidden platform and the results of probe trial reflected the disturbance in spatial memory. XSECC remarkably attenuated these alterations, suggesting that XSECC was able to improve CCH-induced hippocampus-dependent spatial learning and memory impairment.

The chronic reduction of blood flow in the brain closely resembles the condition of reduced CBF in human aging and dementia and contributes to behavioral and cognitive deficits^17^. Therefore, we further used 3D TOF MRA, which is one of the most advanced imaging techniques, to monitor the hippocampal perfusion and the morphologic changes of intracranial arteries *in vivo*^18^. In the present study, our results showed that the signal intensities of ICA, ACA and MCA were significantly decreased and the signal intensities of BA and the PCA were considerably increased after BCCAO. Moreover, BA appeared more tortuous in the model rats compared to the sham control rats, suggesting that BCCAO induced a compensative mechanism attempting to maintain optimal CBF to the brain^19^. However, this compensation after BCCAO may be limited as evidenced by obviously reduced CBF in the hippocampus detected by ASL perfusion MR imaging. The circle of Willis constitutes the main network of collateral circulation and is immediately available to maintain perfusion in case of acute large artery occlusion to prevent brain damage^20^. Following carotid artery occlusion, areas supplied by the ICA might be changed to the vertebral basilar arteries such as PCA^21^. Thus, the CBF in the hippocampus was not sufficient to maintain normal functions. Furthermore, the occlusion of carotid arteries leads to the enlargement of PCA and abnormal tortuosity of BA^19^. The vascular structural abnormalities could impair the cerebrovascular auto-regulatory function and might make it inadequate to adapt CBF metabolic requirements of the hippocampus^22^. These findings indicated that occlusion of both intracranial carotid arteries exerted profound influences on the hippocampal CBF and cerebral vessels which consequently contributed to the hippocampal hypoperfusion^23^. Notably, the hippocampal CBF was elevated in rats with BCCAO after XSECC treatment. In addition, the signal intensities of the bilateral ICA and ACA were increased and the BA showed less tortuous on MRA images, indicating that the hemodynamic effect of XSECC may be associated with activating the circle of Willis.

CCH has been reported to be systemically accompanied by an impaired glucose metabolism in the affected brain regions including hippocampus^24^. The dysfunction of glucose metabolism in the hippocampus may be highly associated with neuronal degeneration and cognitive decline after CCH^21^. Micro-PET was considered to be the most reliable imaging modality to detect the cerebral energy metabolism *in vivo*^25^. In the present study, we used a small animal micro-PET/CT scanner with ^18^F-FDG as a molecular probe to monitor regional cerebral glucose utilization. The SUV of ^18^F-FDG was significantly decreased in the hippocampus of BCCAO rats measured with the FDG-PET technique. However, XSECC treatment elevated the glucose uptake in the hippocampus after BCCAO. Correlative analysis showed that the increased glucose metabolism in the hippocampus was positively correlated with the cognitive performance, suggesting an important role of glucose metabolic abnormality in cognitive impairment induced by CCH. These results indicated that XSECC promoted the glucose metabolism following CCH, which might partially contribute to its cognition-improving properties.

It has been reported that hippocampal neuronal impairment and neuroplasticity damage also play important role in CCH-induced cognitive deficits^26^. In this study, although the T2 data from MRI showed that no infarction were observed in all cerebral areas after BCCAO, the histological studies demonstrated several neuropathological consequences in the hippocampal CA1 region induced by CCH. Morphological analysis revealed that the pyramidal neurons in the CA1 subfield of the hippocampus were damaged after CCH, which were further confirmed by NeuN staining. Moreover, ultrastructural analysis indicated the breakdown of myelin sheath and axon, and immunohistochemical analysis of APP expression revealed obvious axonal injury in the hippocampal CA1 subfield. Consistent with previous studies, our present study suggested that neuronal damage accompanied by axonal degeneration in the hippocampus following CCH^6^. In addition, XSECC treatment ameliorated these pathogenic events, indicating that XSECC may have long-term neurological benefits against hippocampal damage induced by CCH.

Hippocampal neuroplasticity is a critical aspect of brain repair and contributes to the improvement of learning and memory^27^. Evidence supporting the involvement of the neuroplasticity in XSECC treatment was demonstrated in this study. Firstly, the synaptic ultrastructural analysis showed that XSECC treatment significantly increased the number of synapses as well as the length and thickness of PSD in the hippocampal CA1 region of BCCAO rats. The PSD lies on the postsynaptic membrane at the synaptic contact zone, and the structural modifications of PSD are closely related to synaptic plasticity which involved in the processes of learning and memory^28^. Secondly, XSECC treatment markedly up-regulated the expression of neuronal plasticity markers, such as SYN and GAP-43, in the hippocampus after CCH. It has been reported that SYN and GAP-43 are closely related to axonal reorganization and synaptic plasticity in the injured brain. SYN lies on the membrane of presynaptic vesicles and is known as a neuronal marker of synaptogenesis. The high level of SYN has been found to be a sensitive marker of synapse formation^5,29^. GAP-43 is an intracellular growth-associated protein that mainly localizes to the neuronal growth cone membrane. The high level of GAP-43 expression is expected to be associated with axonal extension and cytoskeletal reorganization^27^. Therefore, XSECC modified the synaptic structure and increased the expression of neuronal plasticity markers in the hippocampus after CCH, which might be partially contributed to the cognition-enhancing effects of XSECC.

Given that BYHWT promoted neurological recovery and angiogenesis after intracerebral hemorrhage *via* AKT signaling pathway^30^, we speculated the underlying mechanism of promoting neuroplasticity of XSECC might be associated with AKT signaling. As a survival kinase, AKT has been regarded as an important neuronal protective effector after CCH. The activation of AKT requires the phosphorylation at Ser473 and mediates its downstream target GSK-3β at Ser9, which promotes axonal regeneration and neuronal plasticity after cerebral injury^31,32^. Moreover, CRMP2, a substrate of GSK-3β, is enriched in the growing axon of hippocampal neurons and is critical for neuronal polarity and axonal elongation^33^. GSK-3β decreases the binding activity of CRMP2 to tubulin through phosphorylation of CRMP2, thereby inhibiting microtubule polymerization and stabilization^34^. But the activities of GSK-3β can be inhibited by AKT, eventually renders CRMP2 activity. The current study showed reduced active AKT-GSK-3β phosphorylated at Ser9, concurrent with increased phosphorylation of CRMP2 in hippocampus of rats subjected to BCCAO without treatment. Furthermore, XSECC treatment markedly attenuated CCH-induced increase in the level of phospho-CRMP2, but increased the expression of phosphorylated AKT and GSK-3β, indicating that XSECC had ability to activate AKT signaling pathway, which might, at least partially, contribute to the effect of promoting neurite outgrowth of XSECC following CCH.

Collectively, our study demonstrated that XSECC ameliorated CCH-induced hippocampus-dependent spatial learning and memory impairments by improving hemodynamics and glucose metabolism, as well as promoting neuroplasticity in the hippocampus. This study also elucidated the molecular mechanisms underlying the effects of XSECC on cognitive deficits and brain damages in the BCCAO rat model. Indeed, we found that XSECC treatment could phosphorylate/activate AKT and promote the phosphorylation of GSK-3β, thereby enhancing CRMP2 activation in the hippocampus in a rat model of CCH. Given that AKT/GSK3β-mediated activation of CRMP2 is critical for the axonal regeneration and cognitive function improvement after brain injuries^35^, the protective effects of XSECC on the cognitive function of rats after CCH may involve the regulation of the AKT/GSK-3β/CRMP2 signal pathway.

XSECC is prepared from seven traditional Chinese medicines, including *Radix Astragali*, *Radix Angelicae Sinensis*, *Radix Paeoniae Rubra*, *Rhizoma Ligustici Chuanxiong*, *Flos Carthami*, *Semen Persicae* and *Pheretima*. The pharmacology of XSECC is complex due to its wide range of active constituents such as hydroxysafflor yellow A, paeoniflorin and calycosin-7-O-β-D-glucoside, which have potential neuroprotective properties^9^. Evidence from experimental studies reveals that treating cerebral ischemic rats with calycosin-7-O-β-D-glucoside protects blood-brain barrier integrity and improves hemodynamics through regulating PI3K/AKT signal pathway on cerebral ischemia^36,37^. Hydroxysafflor yellow A can enhance long-term potentiation (LTP) and improve the spatial memory of vascular dementia rats by promoting the expression of brain derived neurotrophic factor (BDNF) and GluN2B (NMDAR) in the hippocampus^38^. Additionally, paeoniflorin ameliorates cognitive deficits through suppressing tau hyperphosphorylation and inflammatory reaction in diabetic rats^39^. Taken together, these studies indicate that the neuroprotective effects of XSECC may rely on the actions of multiple constituents aiming at multiple targets and mechanisms.

Although the present study suggested that the protective effects of XSECC on CCH-induced cognitive impairment in rats were achieved likely via regulating AKT/GSK-3β/CRMP2 signal pathway, we did not exclude the possibility that other cellular and molecular mechanisms were implicated in XSECC-induced improvement of the cognitive function following CCH. Further investigation as to how XSECC impacts the cell-signaling molecules in the amelioration of vascular cognition disturbance is warranted, which may produce clinical benefits such as protection against CCH-induced neurodegeneration and cognitive deficits.

## Materials and Methods

### Animals

Male Sprague-Dawley rats (Grade II, certificate number 118, SCXK [jing] 2012–0001), weighing 180~200 g, were purchased from Vital River Laboratory Animal Technology Co. Ltd. (Beijing, China). The room in which rats were housed was 21 ± 2 °C and 55 ± 5% relative humidity with artificial 12:12 h equivalent light-dark cycles. All experimental procedures were carried out according to the National Institute of Health Guide for the Care and Use of Laboratory Animals, and approved by the Ethical Committee at the Capital Medical University (Permit Number: AEEI-2015-111). Efforts were made to reduce the number of animals used and to avoid their suffering wherever possible.

### Drug

XSECC (batch no. 20140407), supplied by Sanmenxia Sinoway Pharmaceutical Co. Ltd. (Henan, China), was approved by the Chinese State Food and Drug Administration (CFDA) (drug approval number Z20000025). The ingredients of XSECC were carefully analyzed and quality controlled as previously described^15^. The details were described in the Supplementary data. In our study, XSECC was dissolved in physiological saline to make a solution at concentration of 42, 14 and 4.7 mg/ml before experiment.

### CCH model and drug administration

CCH was induced by BCCAO surgical operation as previously described^40^. Briefly, anesthesia was induced by 4% isoflurane vaporized in air and maintained with 2% isoflurane during the surgical procedures. After a ventral midline cervical incision established, bilateral common carotid arteries were carefully separated from the vagal nerves, and then permanently doubly ligated with 4-0 silk sutures. The neck incision was closed with silk sutures. Sham-operated rats were subjected to the same surgical procedure, without occlusion of the bilateral common carotid arteries.

The animals were randomly assigned to the following experimental groups (n = 10 per group): sham surgery group, BCCAO model group, BCCAO plus XSECC (47, 140 and 420 mg/kg) groups, and BCCAO plus EGb 761 (60 mg/kg) group. Rats in the treated groups were orally administrated 24 h after BCCAO and daily thereafter until sacrificed on the 40^th^ day. While the sham-operated and model group rats were treated with normal saline (10 ml/kg/day).

EGb 761 served as positive control and was given a dose of 60 mg/kg according to a previous study, which demonstrated the beneficial effect of this dose on hippocampus-dependent spatial learning and memory and synaptic plasticity of aged rats^41^. According to clinical practical and human-rat equivalent dosage conversion, the administration dosage of XSECC was 140 mg/kg/day, and our previous results indicated that the administration dosage of XSECC by 420 mg/kg/day exhibited maximal protective effects on ischemic injury^9^. Therefore, in this study, the dose of XSECC in the therapy of CCH was set at 47, 140 and 420 mg/kg in order to pick up the effective doses.

### Morris water maze

Morris water maze was performed to evaluate the spatial learning and memory on the 35^th^ day after surgery^42^. The maze consisted of a circular pool with a circular transparent platform (15 cm in diameter). The pool was filled with water (18–20 °C), and divided into quadrants I, II, III, and IV. In the hidden platform test, the platform was hidden 2 cm below the water surface in quadrant I. Rats were given a maximum of 60 seconds to reach the platform. If the rat did not reach the escape platform within 60 seconds, it would be guided to the platform by the experimenter and left on the platform for 10 seconds to help the rats orient to its surroundings. The escape latency and swimming traces of rats in the hidden platform test were monitored and analyzed by a computer-driven movement tracing system. (JLBehv-MWMG, Jiliang Software Technology Co. Ltd., Shanghai).

To assess spatial memory, a spatial probe trial was done 24 h after the end of the hidden platform test. The platform was removed from the maze, and the rats were allowed 30 seconds to search. Time and the path of rats spending in the target quadrant (quadrant I, in which the original platform was located) were analyzed for the probe trial.

### Magnetic resonance imaging

MRI scans were conducted by a 7.0 T animal scanner (Bruker Biospin, Ettlingen, Germany) as previously described^9^. Rats were anesthetized with 5% isoflurane and then maintained with 2.5% isoflurane to ensure complete unconsciousness throughout the scanning process. During the MRI scan, the rectal temperature of rats was kept at 37 ± 0.5 °C with a feedback-controlled warm air system and the respiration of rats was monitored.

T2 relaxometry mapping was acquired to evaluate the brain lesions by using multi-slice multi-echo (MSME) sequence with 16 echo times (TEs), from 11 to 176 ms, repetition time (TR) = 2500 ms, field of view (FOV) = 3.3 × 3.3 cm^2^, and matrix size = 256 × 256^9^. Regions of interest (ROIs) were placed on the bilateral hippocampus according to the Paxinos and Watson atlas^43^. Average of bilateral hippocampus was used as value for T2 value.

3D TOF-MRA was performed to analyze the vascular structure and function using a fast low angle shot sequence (FLASH) with following parameters: TR/TE = 15/2.5 ms, number of matrix = 1, matrix size = 256 × 256 × 128, FOV = 3.5 × 3.5 × 4 cm^3^ ^9^. The signal intensities of the main cerebral arteries were acquired according to a previously described method^44^. The tortuosity of basilar artery (BA) was calculated as the ratio between the hypothetical minimum length and the true length of the BA^19^.

ASL was applied to measure cerebral perfusion by using an echo-planar imaging fluid-attenuated inversion recovery (EPI-FLAIR) sequence^9^. The acquisition parameters were as follows: TR/TE = 18000/25 ms, matrix size = 128 × 128, FOV = 3.0 × 3.0 cm^2^ and the number of excitations = 1. The imaging slice was 1 mm thick (bregma −3.8 mm). ROIs were symmetrically drawn on the bilateral hippocampus in CBF maps to obtain CBF values.

### Micro PET imaging acquisition and data analysis

After MRI scanning, PET imaging was carried out to monitor cerebral glucose metabolism on a high-resolution micro PET/CT scanner (Inveon, Siemens Medical Solution, Germany). Briefly, rats were fasting and water deprivation for 8 h before scanning. Then rats were intravenously injected with 18.5 MBq (500 μCi) of ^18^F-FDG through femoral vein under anesthesia by inhalation of 2.5% isoflurane. After a 60-minute uptake period during which rats were awake, rats were placed in the prone position on the examination bed, with 2.5% isoflurane continuously carried by breath mask to ensure animal remain completely unconscious. The scanning procedure consisted of a twenty-minute static acquisition period and an eight-minute attenuation-correction CT scan. All images were reconstructed using a two-dimensional ordered-subset expectation maximum (2D OSEM) algorithm and analyzed by Inveon Research Workplace software. Experimenter blinded to experimental design manually selected ROI of hippocampus on PET/CT fusion images^45^. SUV of ^18^FDG in the bilateral hippocampus was calculated as the regional radioactivity concentration normalized by the total injected dose and body weight^46^.

### Tissue preparation, histological and immunohistochemical observation

At the end of the PET imaging, rats were deeply anesthetized and perfused transcardially with 0.9% saline followed by 4% paraformaldehyde in 0.1 m/L phosphate-buffered solution (PBS, pH 7.4). The brain tissues were then removed and post-fixed in the same fixative at 4 °C overnight. A standard paraffin block from the lesion (bregma −2.92 to −3.72 mm) were embedded in paraffin, 4 µm thick sections from the block were made and processed.

HE staining was performed to observe morphological characteristics of neuronal perikarya as reported previously^47^. NeuN immunofluorescence staining was performed to identifiy damages to neurons as described previously^10^. Briefly, coronal brain sections were incubated at 4 °C for 40 h with NeuN antibody (1:200, Millipore) followed with Alexa Fluor 488 secondary antibody for 2 h at 37 °C. The sections were mounted onto slides, coverslipped with DAPI. APP immunohistochemical staining was conducted to detect axonal injury as described previously^10^. Briefly, the sections were incubated with APP antibody (1:200, EPITOMICS) for 40 h at 4 °C followed by incubation with biotinylated goat anti-rabbit IgG secondary antibody (Zhongshan-Golden Bridge, Beijing, China) at 37 °C for 60 min. Slices were visualized through DAB kit (Zhongshan-Golden Bridge, Beijing, China).

For quantitative analysis of HE, NeuN and APP staining, four non-overlapping fields (400× magnification) within hippocampal CA1 of each section were taken by a digital camera connected to an optical and a fluorescence microscopy (Nikon 80i, Nikon, Tokyo, Japan) and processed using NIS-Elements Basic Research Image Collection Analysis system (Nikon, Japan). The stereological method was applied to count surviving pyramid cells in the hippocampus (CA1) of HE staining images. In brief, the number of neurons was obtained from different vision fields of the hippocampal CA1 and data were expressed as the mean number of pyramid cells per mm^2^ ^48^. The expression levels of NeuN and APP were expressed as integrated optical density (IOD) values as previously described^49^. All the analysis was conducted by an investigator blinded to the groupings.

### Transmission electron microscopy

TEM was used to observe the ultrastructural changes in the hippocampal CA1 region. Briefly, the hippocampus CA1 region was carefully separated and rapidly cut into small pieces (1 mm^3^) and then transferred to the fixative buffer for 2 h. After fixation, samples were dehydrated in graded acetone and embedded in Epon812. The ultrathin sections were stained with 4% uranyl acetate-lead citrate and examined by TEM (JEM-1230, Jeol, Japan). The synapse was identified based on a criteria described previously^50^, and the synaptic density was expressed as the number of synapses per 100 μm^2^ ^26^. In addition, the thickness (the thickest part of PSD) and length of PSD were measured using ImageJ (NIH) software^50^.

### Western blot analysis

Western blot was conducted as previously described^15^. Briefly, after removal of the brains, the bilateral hippocampal CA1 tissues were homogenized in ice-cold RIPA protein extraction buffer (Pulilai, China) containing 1% protease and phosphatase inhibitors. Equal amounts of protein were subjected to 10% SDS-polyacrylamide gel electrophoresis (PAGE) and then electro-transferred to polyvinylidene fluoride (PVDF) membranes (0.45 μm). After blocking with 5% nonfat milk or 5% BSA, target proteins were detected using the following primary antibodies: anti-APP (1:20000, EPITOMICS), anti-growth associated protein-43 (GAP-43, 1:50000, EPITOMICS), anti-synaptophysin (SYN, 1:50000, EPITOMICS), anti-p-AKT (Ser473, 1:5000, Cell signaling), anti-AKT (1:20000, Cell signaling), anti-p-GSK-3β (Ser9, 1:1000, Cell signaling), anti-GSK-3β (1:5000, Abcam), anti-p-CRMP2 (Thr514, 1:100000, Abcam), anti-CRMP2 (1:50000, Abcam), and anti-GAPDH (1:20000, Neobioscience). Secondary antibodies were incubated for 1 h at 37 °C. The immunoreactive bands were detected using enhanced chemiluminescence detection kit (Millipore, USA) by exposure to X-ray films, and then analyzed using Image J software. Protein level was normalized to the matching densitometric value of the internal control GAPDH.

### Statistical analysis

All data were expressed as mean ± standard error of the mean (SEM). The statistical analyses were performed using the SPSS 19.0 (SPSS Inc., USA) software. Escape latency and path length in Morris water maze test was analyzed by two-way ANOVA with repeated measures followed by Turkey’s *post hoc* tests. Other multiple comparisons were performed by one-way ANOVA followed with LSD’s *post hoc* tests. Pearson correlations were calculated to examine the linear association between hippocampal SUV of ^18^FDG and Morris water maze performance in the probe test. Values of *P* < 0.05 were considered statistically significant.

## Electronic supplementary material

Supplementary information
