# Supplementary material for: Xiaoshuan enteric-coated capsule alleviates cognitive impairment by enhancing hippocampal glucose metabolism, hemodynamics and neuroplasticity of rat with chronic cerebral hypoperfusion
Source: Sci Rep. 2018 May 10;8:7449. doi: 10.1038/s41598-018-25929-0 (PMC5945608; doi:10.1038/s41598-018-25929-0)
Supplement: Supplementary file 1 — Supplementary information [file 41598_2018_25929_MOESM1_ESM.pdf]

Xiaoshuan enteric-coated capsule alleviates cognitive impairment by enhancing hippocampal glucose metabolism, hemodynamics and neuroplasticity of rat with chronic cerebral hypoperfusion

Man-zhong Li[1,2,#], Yi Zhang[3,#], Hai-yan Zou[1,2], Ya-li Wang[1,2], Brian-Chi Yan Cheng[4], Lei Wang[1,2], Qiu-xia Zhang[1,2], Jian-feng Lei[5], Hui Zhao[1,2,*]

[1]School of Traditional Chinese Medicine, Capital Medical University, Beijing 100069, China.

[2]Beijing Key Lab of TCM Collateral Disease Theory Research, Beijing 100069, China.

[3]Department of pharmacology, Beijing University of Chinese Medicine, Beijing 100102, China.

[4]College of Professional and Continuing Education, The Hong Kong Polytechnic University, Hung Hom, Hong Kong.

[5]Medical Imaging laboratory of Core Facility Center, Capital Medical University, Beijing 100069, China.

[*]Corresponding author.

E-mail address: zhaohuishouyi@sina.com (H. Zhao).

[#]The two authors contributed equally to this work.

Supplementary data

Chemicals

Hydroxysafflor yellow A (batch no. MUST-13121913), paeoniflorin (batch no. MUST-13113009), calycosin-7-O-β-D-glucoside (batch no. MUST-14031314), calycosin (batch no. MUST-13081501), and formononetin (batch no. MUST-14030710) were purchased from Chengdu Must Bio-technology Co. Ltd. (Sichuan, China). Ononin (batch no. 130507) was obtained from Chengdu Pufei De Biotech Co. Ltd. (Sichuan, China). The purity of all the above chemical reference substances was determined to be higher than 98%.

Quantitative analysis of XSECC by HPLC

The contents of six main components in XSECC, including hydroxysafflor yellow A, paeoniflorin, calycosin-7-O-β-D-glucoside, ononin, calycosin and formononetin (Supplementary **Fig. S1**), were determined using an Agilent 1260 liquid chromatography system (Agilent Technologies, USA), consisted of an autosampler, a quaternary pump, a diode-array detector, and a column temperature controller. Agilent Chem Station software version B.04.03C was used for data collection and analysis. Before the chromatographic analysis, 0.4 g capsule powder was ultra-sonicated (300 W, 40 kHz) with 50% methanol (4 mL) at 30 ℃ for 30 min, then dilute with 50% methanol to 5 mL and filtrated through 0.45 μm membrane filter. The analysis was performed on a Zorbax SB-C18 (250 mm×4.6 mm, 5 μm, Agilent) column at a temperature of 30℃, using a gradient mobile phase consisting of acetonitrile (solvent A) and 0.1% phosphoric acid (v/v, solvent B). The linear gradient was as follows: 0 - 15 min, 99 - 90% B; 15 - 45 min, 90 - 60% B; 45-60 min, 60 - 30% B; 60-80 min, 30 - 0% B. The flow rate was 1.0 mL/min. The wavelength was set at 230 nm (for paeoniflorin), 254 nm (for calycosin-7-O-β-D-glucoside, formononetin, calycosin and ononin) and 403 nm (for hydroxysafflor

yellow A). The injection volume was 5 μL. Under the current conditions, HPLC peaks for all the quanlity control components could be clearly separated. (Supplementary **Fig. S2**). The contents of hydroxysafflor yellow A, paeoniflorin, calycosin-7-O-β-D-glucoside, formononetin, calycosin and ononin were 1.24 mg/g, 2.12 mg/g, 1.04 mg/g, 0.42 mg/g, 0.13 mg/g and 0.08 mg/g, respectively.

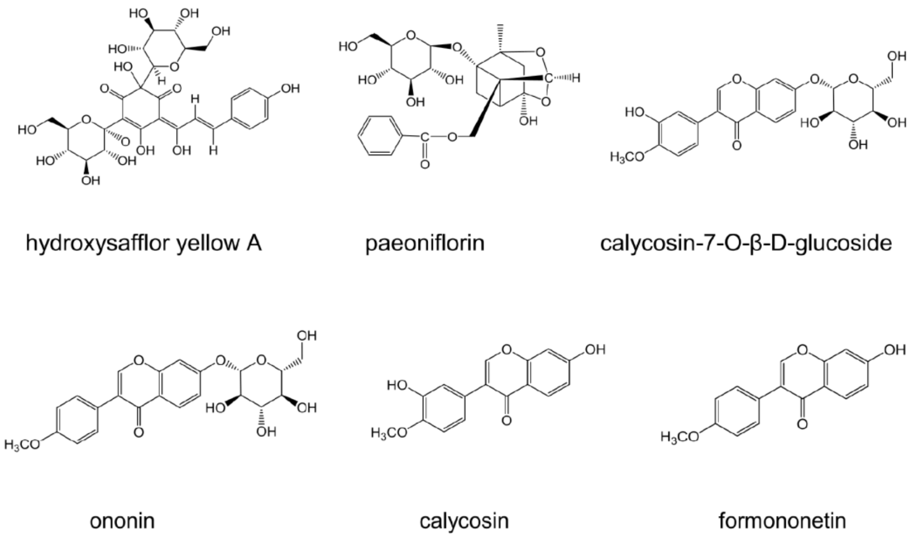

Supplementary Figure S1. The chemical structures of six major components identified from XSECC.

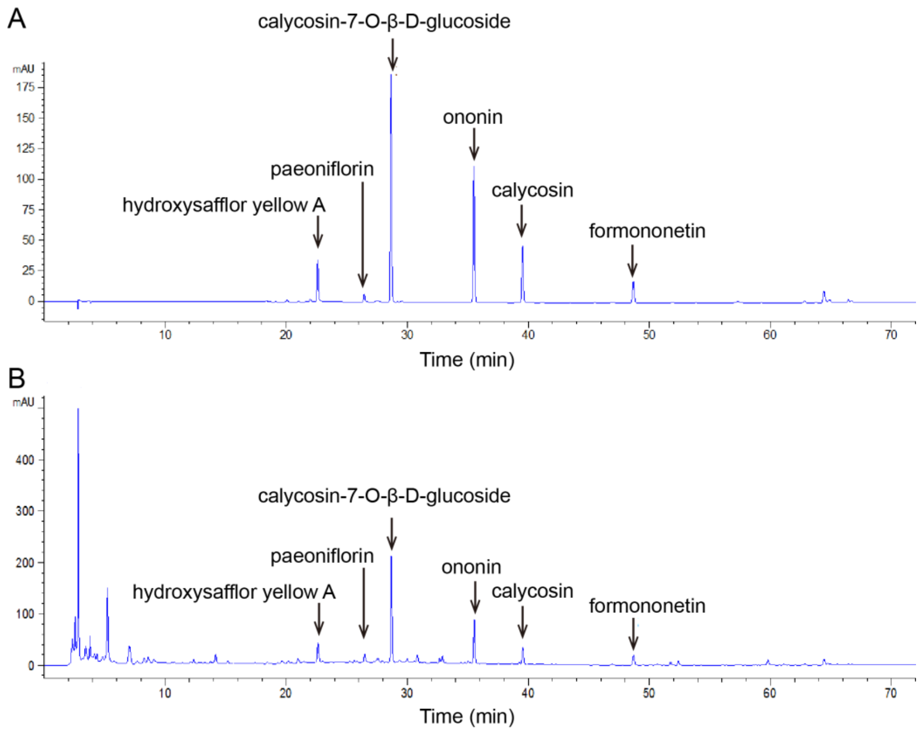

Supplementary Figure S2. The HPLC chromatograms of mixed standards (A) and XSECC (B) at 203 nm.

Uncropped images of western blotting

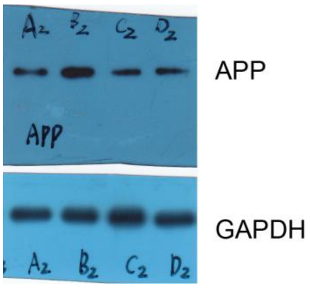

Supplementary Figure S3. The original western blots depicted in Figure 5. (A, sham; B, model; C, XSECC 420 mg/kg; D, XSECC 140 mg/kg)

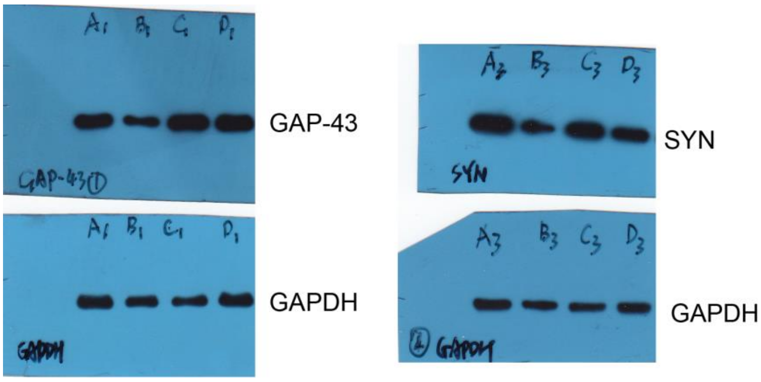

Supplementary Figure S4. The original western blots depicted in Figure 6. (A, sham; B, model; C, XSECC 420 mg/kg; D, XSECC 140 mg/kg)

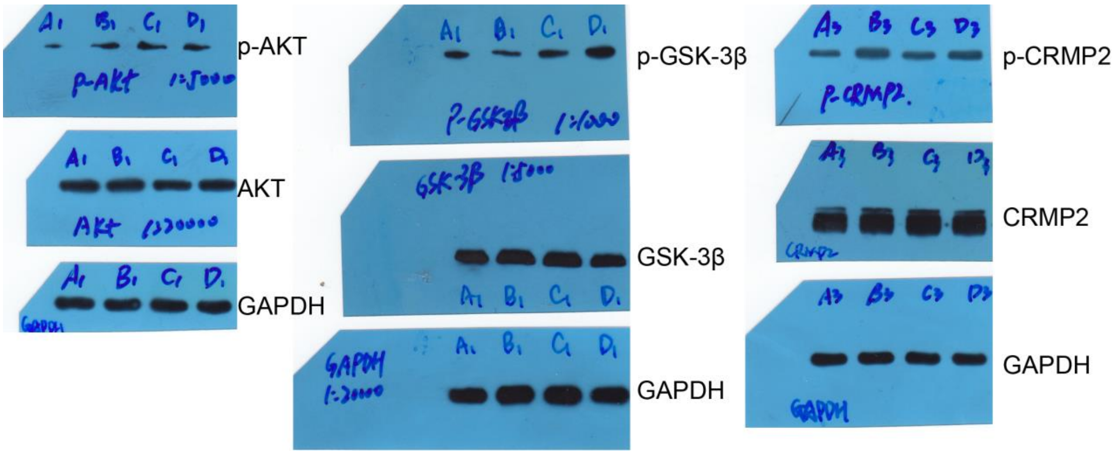

Supplementary Figure S5. The original western blots depicted in Figure 7. (A, sham; B, model; C, XSECC 420 mg/kg; D, XSECC 140 mg/kg)
